# Supplementary material for: The Roles of Different Fractions in Freshwater Biofilms in the Photodegradation of Methyl Orange and Bisphenol A in Aqueous Solutions
Source: Int J Environ Res Public Health. 2022 Oct 11;19(20):12995. doi: 10.3390/ijerph192012995 (PMC9601981; doi:10.3390/ijerph192012995)
Supplement: Supplementary file 1 [file ijerph-19-12995-s001.zip › ijerph-1936381-supplementary.pdf]

The Roles of Different Fractions in Freshwater Biofilms in the Photodegradation of Methyl Orange and Bisphenol A in Aqueous Solutions

Haojie Yin [1,2], Lingling Wang [1], Guangshu Zeng [2], Longfei Wang [1,2,*] and Yi Li [2,*]

[1] Institute of Microbiology, Guangdong Academy of Sciences, State Key Laboratory of Applied Microbiology Southern China, Guangzhou 510070, China
[2] Key Laboratory of Integrated Regulation and Resource Development on Shallow Lakes, Ministry of Education, College of Environment, Hohai University, Nanjing 210098, China
* Correspondence: lfwang@hhu.edu.cn (L.W.); envly@hhu.edu.cn (Y.L.)

Table S1 The water qualities at the sites for sampling River Biofilm and Pond Biofilm.

Parameters	Numerical values for sampling site collecting River Biofilm	Numerical values for sampling site collecting Pond Biofilm	Unit
pH	7.41	7.08	-
Conductivity	343.00	368.00	μS/cm
Redox potential	-59.90	-47.30	mV
COD	23.45	17.62	mg/L
Ammonia	1.18	1.03	mg/L
TP	0.12	0.10	mg/L
TN	9.66	1.57	mg/L

Table S2 Direct photodegradation rates of pollutants at a concentration of 2 mg/L.

**Condition**	**k(10^{-2}min^{-1})**	**R^2**
MO	0.166±0.033	0.79
MO+348 mg/L pond biofilm suspension	0.231±0.015	0.98
MO+696 mg/L pond biofilm suspension	0.458±0.062	0.90
MO+348 mg/L river biofilm suspension	1.181±0.108	0.95
MO+696 mg/L river biofilm suspension	1.552±0.199	0.91
BPA	0.041±0.008	0.78
BPA+348 mg/L pond biofilm suspension	0.043±0.006	0.89
BPA +696 mg/L pond biofilm suspension	0.093±0.017	0.82
BPA +348 mg/L river biofilm suspension	0.168±0.014	0.96
BPA +696 mg/L river biofilm suspension	0.303±0.017	0.98

Table S3 Effects of initial pollutant concentration to photodegradation behaviors.

Initial concentration of MO (mg/L)	k (10^{-2}min^{-1})	R^2	Initial concentration of BPA (mg/L)	k (10^{-2}min^{-1})	R^2

1	1.806±0.156	0.96	1	0.208±0.006	0.99
2	1.602±0.290	0.83	2	0.228±0.020	0.96
5	0.373±0.106	0.66	5	0.175±0.001	0.97

Table S4 The compositions of EPS derived from periphytic biofilms (EPS content: 50 mgC/L).

	River Biofilm	Pond Biofilm
Carbohydrate (mg/L)	94.3±16.4	72.6±27.5
Proteins (mg/L)	13.2±0.2	4.8±1.0
Humic substances (mg/L)	26.7±0.1	15.5±0.7

Table S5 Spectral parameters of EPS derived from periphytic biofilms.

EPS source	TOC（mgC/L）	a(355)	SUAV254	SUAV260	E_2/E_3	FI	BIX
River Biofilm	50	35.98	2.13	1.85	2.75	1.88	0.98
Pond Biofilm	50	12.87	1.97	1.93	2.52	2.84	1.73

Table S6. The roles of different fractions in biofilms on the direct photodegradation rates during photodegrading MO.

Experimental condition	$k(10^{-2}min^{-1})$	R^2
MO	0.180±0.033	0.83
MO+River biofilm	1.576±0.221	0.89
MO+River biofilm without EPS	0.734±0.071	0.95
MO+River biofilm EPS	0.374±0.043	0.92
MO+Pond biofilm	0.458±0.060	0.90
MO+Pond biofilm without EPS	0.452±0.054	0.92
MO+Pond biofilm EPS	0.176±0.035	0.80

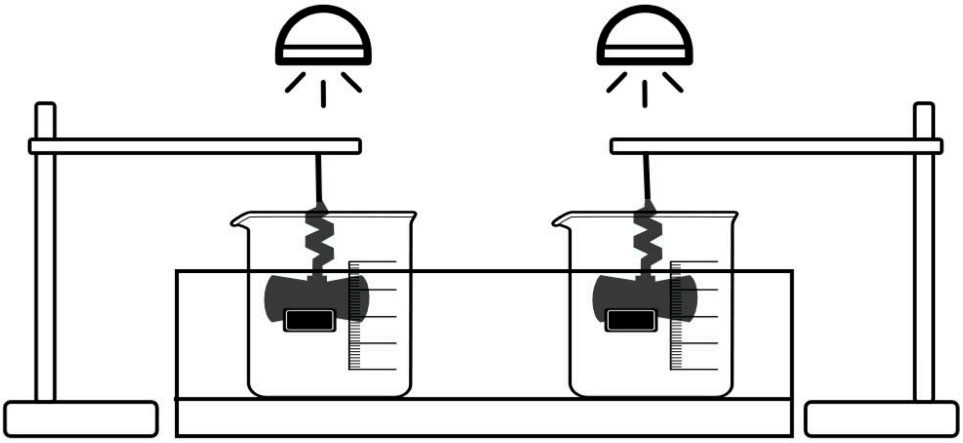

Figure S1. The illustration of the modeled photochemical reactor.

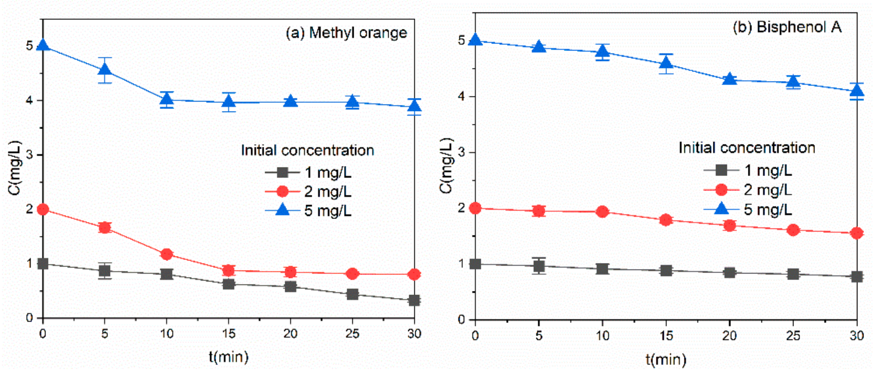

Figure S2 Effects of initial pollutant concentration to photodegradation behaviors of (a) MO and (b) BPA.

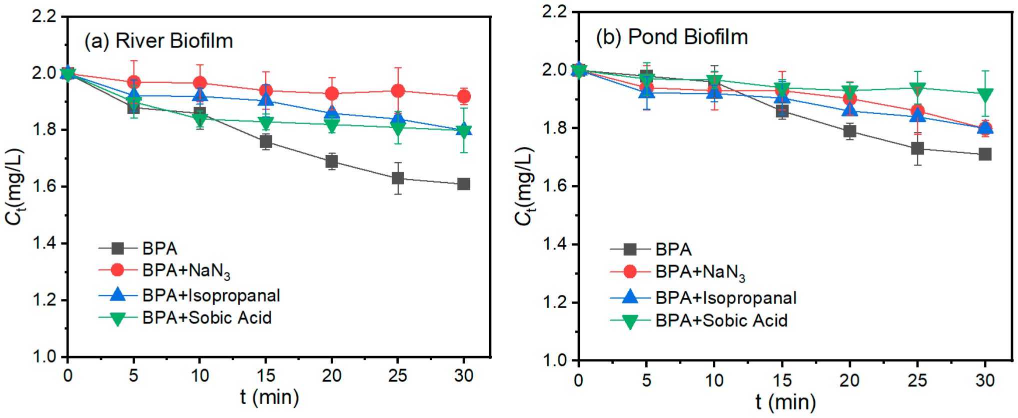

Figure S3. Photodegradation rate of BPA with quenchers (isopropanol, NaN_3 and sorbic acid) in the presence of the suspensions derived from (a) River Biofilm and (b) Pond Biofilm. Experimental conditions: $[MO]_0$ =2 mg/L, [Biofilm]= 696 mg/L, pH = 6.8 (10 mM phosphate buffered).

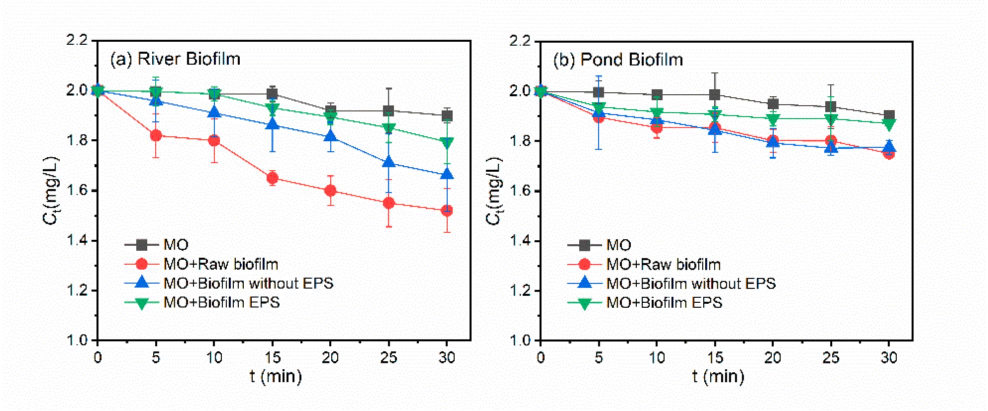

Figure S4 Photodegradation behaviors of BPA in the presence of different biofilm fractions, i.e., raw biofilm at a content of 696 mg/L, the same content of raw biofilm after EPS extraction, as well as the extracted EPS (~14 mgC/L TOC). C_0[BPA]=2 mg/L.

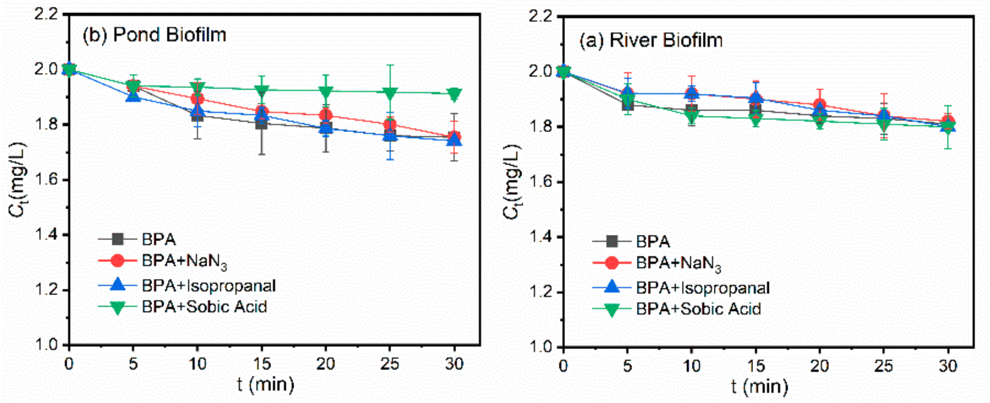

Figure S5. Photodegradation rate of BPA with quenchers (isopropanol, NaN3 and sorbic acid) in the presence of the EPS derived from (a) river biofilm and (b) pond biofilm. Experimental conditions: $[MO]_0$ =2 mg/L, [EPS content]= 14 mgC/L, pH = 6.8 (10 mM phosphate buffered).
